# Mechanosensitive cation channel Piezo1 contributes to ventilator-induced lung injury by activating RhoA/ROCK1 in rats

**DOI:** 10.1186/s12931-021-01844-3

**Published:** 2021-09-21

**Authors:** Yang Zhang, Lulu Jiang, Tianfeng Huang, Dahao Lu, Yue Song, Lihui Wang, Ju Gao

**Affiliations:** 1 Department of Anesthesiology, The Second Xiangya Hospital, Central South University, Changsha, Hunan China; 2 Department of Anesthesiology, Institute of Anesthesia, Emergency and Critical Care, Yangzhou University Affiliated Northern Jiangsu People’s Hospital, 98 Nan Tong Western Road, Yangzhou, 225001 Jiangsu China

**Keywords:** Piezo1, Ventilator-induced lung injury, RhoA/ROCK1, Acute lung injury

## Abstract

**Background:**

Mechanical ventilation can induce or aggravate lung injury, which is termed ventilator-induced lung injury (VILI). Piezo1 is a key element of the mechanotransduction process and can transduce mechanical signals into biological signals by mediating Ca^2+^ influx, which in turn regulates cytoskeletal remodeling and stress alterations. We hypothesized that it plays an important role in the occurrence of VILI, and investigated the underlying mechanisms.

**Methods:**

High tidal volume mechanical ventilation and high magnitude cyclic stretch were performed on Sprague–Dawley rats, and A549 and human pulmonary microvascular endothelial cells, respectively, to establish VILI models. Immunohistochemical staining, flow cytometry, histological examination, enzyme-linked immunosorbent assay, western blotting, quantitative real-time polymerase chain reaction and survival curves were used to assess the effect of Piezo1 on induction of lung injury, as well as the signaling pathways involved.

**Results:**

We observed that Piezo1 expression increased in the lungs after high tidal volume mechanical ventilation and in cyclic stretch-treated cells. Mechanistically, we observed the enhanced expression of RhoA/ROCK1 in both cyclic stretch and Yoda1-treated cells, while the deficiency or inhibition of Piezo1 dramatically antagonized RhoA/ROCK1 expression. Furthermore, blockade of RhoA/ROCK1 signaling using an inhibitor did not affect Piezo1 expression. GSMTx4 was used to inhibit Piezo1, which alleviated VILI-induced pathologic changes, water content and protein leakage in the lungs, and the induction of systemic inflammatory mediators, and improved the 7-day mortality rate in the model rats.

**Conclusions:**

These findings indicate that Piezo1 affects the development and progression of VILI through promotion of RhoA/ROCK1 signaling.

## Background

Mechanical ventilation (MV) is a common method of respiratory support during clinical anesthesia. In many critical diseases, especially acute lung injury (ALI) and acute respiratory distress syndrome (ARDS), MV is an important means of first aid and respiratory treatment. However, in recent years, people have gradually realized that MV is a double-edged sword. MV itself can also induce or aggravate the injury of important target organs. For example, the incidence of ventilator-induced lung injury (VILI), also known as ventilator-associated lung injury [1, 2], accounted for 22%-39% of MV, which could be as high as 83% in patients with lung diseases [3]. Unfortunately, ALI and ARDS-related mortality is very high, and prevention or treatment measures are still limited, possibly because of their complex and unclear pathogenesis. Therefore, understanding the pathological mechanisms of lung injury is imperative to develop prevention and treatment strategies for ALI.

Piezo1 is a mechanosensitive ion channel protein in mammals that can be directly activated by mechanical stimuli, and can transduce mechanical signals into biological signals by mediating Ca^2+^ influx, which in turn regulates vascular development, erythrocyte volume, and urothelial cell tone [4–6]. Endothelium-expressed Piezo1 can sense disturbed blood flow and is linked to inflammatory signaling and atherosclerosis progression [7]. As a specific mechanosensitive protein, whether Piezo1 plays an important role in the occurrence of VILI is not clear.

RhoA is a small GTPase protein in the Rho family that is primarily associated with cytoskeleton regulation, mostly actin organization and actomyosin contractility [8]. Our previous study found that the RhoA/Rho associated coiled-coil containing protein kinase (ROCK) signaling pathway was activated and the expression levels of its members was significantly upregulated in the lung tissues of septic rats [9]. Similarly, it was reported that RhoA activation is involved in lipopolysaccharide-mediated endothelial barrier dysfunction in ALI mice [10]. Further study showed that inhibition of RhoA could rescue high ventilation and lipopolysaccharide induced lung injury significantly [11, 12]. All the above studies confirmed the critical role of the RhoA/ROCK signaling pathway in ALI; however, the upstream regulatory mechanisms are not fully determined and require further study.

In the present study, the role of Piezo1 in VILI was investigated. The findings of both in vivo and in vitro experiments demonstrated that Piezo1 expression is required for the high tidal volume ventilation-induced lung injury in rats and this process likely acts through regulating the RhoA/ROCK pathway.

## Methods

### Animal preparation

Adult male Sprague–Dawley rats (250–300 g) were purchased from the Animal Center of the School of Medicine, Yangzhou University. The rats were housed in air-filtered rooms and were given ad libitum access to food and water. Animals were housed at a constant temperature (20–24 °C) and constant humidity (50%–70%) with a 12/12 h light/dark cycle. The study protocol was approved by the Animal Care and Use Committee of Yangzhou University (Yangzhou, China) and was in accordance with the guidelines for the care and use of animals set by the Chinese government.

### Experimental procedure and animal model of VILI

Lung injury was induced in the rats using high tidal volume mechanical ventilation (HVMV), based on a previously published VILI model [13]. Briefly, all animals were anesthetized via an intraperitoneal injection of pentobarbital sodium (40 mg/kg, Merck, Darmstadt, Germany). After induction of anesthesia, the rats underwent an oral endotracheal intubation with a 16 G trocar and were ventilated for hours in a volume-controlled ventilation mode (DW 3000, Zhenghua Biologic, Anhui Province, China). Animals were ventilated with a high tidal volume (Vt) of 22 mL/kg and zero positive end-expiratory pressure (PEEP) at a respiratory rate of 16–18 breaths/min, whereas control (sham) rats were ventilated with a Vt of 6 mL/kg and PEEP of 5 cmH_2_O at a rate of 45–55 breaths/min. The fraction of inspired oxygen (FiO_2_) remained constant at 0.21. The rats were placed in the supine position on a heating blanket and under a heating lamp, to ensure a body temperature of 37 °C throughout the experiment. A polyethylene catheter was placed in the femoral artery to monitor mean arterial pressure and heart rate, as well as for blood sampling. The arterial catheter was infused with physiological saline at 0.5 mL/h and anesthesia was maintained by additional injections (15 mg/kg, i.p.) administered every hour under hemodynamic monitoring. After MV, the rats were returned to their cages and provided food and water ad libitum.

All animals receiving different ventilation times were allocated into 4 groups using a random number table, with six rats per group in sham, HVMV 2, 4 and 6 h. And another 88 rats were equally assigned to sham + vehicle, VILI + vehicle, GSMTx4 + VILI and GSMTx4 + sham these four groups. VILI model group animals received HVMV for 6 h. GSMTx4, an inactive inhibitor of non-selective cationic mechanosensitive channels (MSCs) [14], which was used to inhibit Piezo1 channel activity. 10 μg of GSMTx4 (dissolved in 0.2 mL of saline) or vehicle was injected via arterial supply of the hindlimb thirty minutes before the VILI procedure. Rats were killed by heart bloodletting 6 h after administration of ventilation.

### Cell culture and transfection

The human alveolar epithelial cell line (A549) and the human pulmonary microvascular endothelial cell line (HPMEC) were purchased from the BNCC Biotechnology Research Institute (Beijing, China). The cells were cultured in Dulbecco’s modified Eagle’s medium (DMEM) and M199 (HyClone, Logan, UT, USA) supplemented with 10% fetal bovine serum (Gibco, Carlsbad, CA, USA), 100 U/mL penicillin, and 100 μg/mL streptomycin at 37 °C in an atmosphere of 95% air and 5% CO_2_. When the cells reached 80% confluence, they were seeded into 24-well or 6-well plates for further experiments. For small interfering RNA (siRNA) transfection, the *Piezo1* siRNA (Thermo Fisher Scientific, Waltham, MA, USA) and its negative control siRNA (Invitrogen) were transfected into the cells using Lipofectamine 3000 (Invitrogen, Carlsbad, CA, USA) according to the manufacturer’s instructions. Three days later, the cells were collected. HPMEC monolayers were pre-treated with different concentration of Yoda1 (Tocris/BioTechne, Bristol, UK), 5 μM GSMTx4, 10 μM Y-27632 or fasudil (Merck Millipore, Burlington, MA, USA) for 24 h before cell deformation or collection.

### Cell deformation

Cell deformation was achieved by stretching with a Flexcell Tension Plus™ FX-4000T system (Flexcell International, Burlington, NC, USA) equipped with a loading station, which was designed to provide uniform strain to the cultured cells. The vacuum pressure was controlled by the computer, allowing cell monolayers to receive different levels of elongation. These deformations were selected as previously described [15]. Briefly, cells were seeded at 2.0 × 10^5^ cells/cm^2^ on type I collagen-coated flexible bottom of BioFlex plates (Flexcell international) and allowed to reach 50% confluence after 24 h. Then, the culture medium was changed to serum-deprived medium in each plate and the experimental plates with monolayer cell were mounted onto the Flexcell system. Cells were then exposed to cyclic stretch (CS) of high magnitude (20% elongation) for different durations (0–6 h) with a frequency of 15 cycles/min.

### Flow cytometric analysis of cell apoptosis

To investigate the time-dependent effects of CS on cell apoptosis, the cells after CS exposure were stained with FITC-conjugated Annexin V and propidium iodide (PI) following the manufacturer’s instructions (KeyGEN Biotech Co. Ltd, China) and was analyzed by flow cytometry (Beckman Coulter Co, USA).

### Determination of water content and histological examination

To evaluate the severity of lung injury, ventilator-induced pulmonary edema was assessed based on the wet-to-dry weight ratio of the lung. The right upper lobe of each lung was weighed immediately after extraction and placed in a 60 °C oven for 72 h. The dried tissue was then weighed to determine the wet-to-dry weight ratio. Samples from the inferior lobe of the right lung were fixed in 4% paraformaldehyde solution, dehydrated sequentially in 50%–100% alcohol, and treated with xylene solution. Then, the tissues were embedded in paraffin, sectioned (thickness, 6 μm), and stained with hematoxylin and eosin (H&E). The samples were assigned an injury score for each of these four categories: Alveolar and interstitial edema, microhemorrhage, inflammatory infiltration, and microatelectasis or alveolar overdistension. The injury scores were assigned as follows: 0, absent with normal appearance; 1, slight; 2, intermediate; and 3, severe [9, 16, 17]. The lung injury score was calculated by adding the individual injury scores for each category. The scoring was performed by a pathologist who was blinded to the data, using a light microscope (× 40, Olympus, Tokyo, Japan) to view the stained tissue samples.

### Protein leakage from capillaries

Pulmonary microvascular permeability was determined using the Evans blue dye extravasation method at 6 h after MV. Evans blue dye (30 mg/kg, Sigma-Aldrich, St. Louis, MO, USA) was administered intravenously at 30 min before the rats were sacrificed. Lungs were perfused to remove blood and extracted. The dye content in the lung tissue was determined spectrophotometry at an optical density of 620 nm [18].

### Immunofluorescence

After animals were deeply anesthetized with pentobarbital sodium, they were perfused with 100–300 mL of 4% paraformaldehyde in 0.1 M phosphate buffer (pH 7.4). The lung was harvested and post-fixed at 4 °C for 24 h. The lung tissues were embedded in Tissue-Tek OCT compound (SAKURA, Tokyo, Japan) and frozen in liquid nitrogen for the preparation of cryosections. Frozen lung tissues were cut to a thickness of 20 μm. After being blocked with phosphate-buffered saline (PBS) containing 10% goat serum and 0.3% Triton X-100 for 1–2 h at 37 °C, the sections were incubated overnight at 4 °C with rabbit anti-Piezo1 (1:300, ProteinTech Group, Rosemont, IL, USA). The sections were then incubated with goat anti-rabbit IgG conjugated with Cy3 (1:500, Jackson ImmunoResearch, West Grove, PA, USA) for 1 h at room temperature. The sections were finally mounted using Vectashield plus 4', 6-diamidino-2-phenylindole (DAPI) mounting medium (Vector Laboratories, Burlingame, CA, USA). HPMECs were fixed in 4% paraformaldehyde, and then incubated with anti-Piezo1 antibody (1:400) overnight at 4 °C. After washing five times with PBS, the cells were incubated with Cy3 (1:500) for 1 h at room temperature. Then, the cells were washed with PBS again five times for 1 h before being stained using DAPI for 2 min. After three further washes, the dishes were observed under a fluorescence microscope. All images were observed using a Leica DMI4000 fluorescence microscope and captured with a DFC365FX camera (Leica, Wetzlar, Germany).

### Enzyme-linked immunosorbent assay (ELISA)

Bronchoalveolar lavage fluid (BALF) was collected and centrifuged at 6 h after MV was performed. The concentrations of tumor necrosis factor alpha (TNF-α), interleukin (IL)-1β, IL-6, and monocyte chemotactic protein 1 (MCP-1) were measured using a commercially available ELISA kit according to the manufacturer’s instructions (R&D Systems, Minneapolis, MN, USA).

### Western blotting analysis

Lung tissues were homogenized and the cultured cells were ultrasonicated in chilled lysis buffer (10 mM Tris, 1 mM phenylmethylsulfonyl fluoride, 5 mM MgCl_2_, 5 mM EGTA, 1 mM EDTA, 1 mM DTT, 40 μM leupeptin, 250 mM sucrose). Approximately 10% of the homogenates (by volume) were used to determine total protein levels. The remained was centrifuged at 4 °C for 15 min at 1000 × *g*. The supernatant was collected as cytosolic proteins. After the concentrations of the proteins were measured using a Bio-Rad protein assay (Bio-Rad, Hercules, CA, USA), equal amounts of total proteins were heated at 99 °C for 5 min and loaded onto a 4%–15% stacking/7.5% separating SDS–polyacrylamide gel (Bio-Rad). The proteins were then electrophoretically transferred onto a polyvinylidene difluoride membrane (Bio-Rad). The membrane was blocked for 2 h at room temperature, and then incubated at 4 °C overnight with the following primary antibodies: rabbit anti-Piezo1 (1:1000; ProteinTech Group), rabbit anti-RhoA (1:5000; Abcam, Cambridge, MA, USA), rabbit anti-ROCK1 (1:1000; Abcam), and rabbit anti-glyceraldehyde-3-phosphate dehydrogenase (GAPDH; 1:2000; Sigma). The proteins were detected using horseradish peroxidase-conjugated anti-rabbit secondary antibody (1:3000; Jackson ImmunoResearch), and exposed using the ChemiDoc XRS System with Image Lab software (Bio-Rad). The intensity of immunoreactive protein bands was quantified using densitometry with the Image Lab software (Bio-Rad).

### RhoA activity assay

Active GTP-bound RhoA was detected in lysates collected from cells subjected to a pull-down assay using a RhoA activation assay kit (Abcam) according to the manufacturer’s indications. Briefly, supernatants were incubated with an anti-active RhoA rabbit monoclonal antibody and protein A/G agarose bead slurry at 4 °C (× 1 h) on a rotator. Bead-precipitated proteins were fractionated and immunoblotted using antibodies against RhoA.

### Quantitative real-time polymerase chain reaction (PCR)

Lung tissues and cells were collected rapidly and pooled together to achieve sufficient RNA. Total RNA was extracted using a miRNeasy kit (Qiagen, Valencia, CA, USA) according to the manufacturer’s instructions. Reverse-transcription to cDNA was achieved using ThermoScript Reverse Transcriptase (Invitrogen/Thermo Fisher Scientific) with oligo (dT) primers (Invitrogen/Thermo Fisher Scientific). The cDNA was then used in a quantitative real-time PCR amplification consisting of 30 s at 95 °C, 30 s at 60 °C, and 30 s at 72 °C for 40 cycles. GAPDH was used as an internal control. Relative changes of mRNA levels were calculated by using the △Ct method (2^−△△Ct^). The primers used in this study were as follows:

*Piezo1* Forward: 5’-GGACTCTCGCTGGTCTACCT-3’;

*Piezo1* Reverse: 5’-GGGCACAATATGCAGGCAGA-3’;

*ROCK1 *Forward: 5’-GACTGGGGACAGTTTTGAGAC-3’;

*ROCK1* Reverse: 5’-GGGCATCCAATCCATCCAGC-3’;

*Tubulin *Forward: 5’-GCCTTCTGAGAGAGTTAAG-3’;

*Tubulin* Reverse: 5’-AGACTGGACCACCGGAGA-3’;

Mouse-*GAPDH* Forward: 5’-AATGGATTTGGACGCATTGGT-3’;

Mouse-*GAPDH* Reverse: 5’-TTTGCACTGGTACGTGTTGAT-3’;

Human-*GAPDH* Forward: 5’-AATGGACAACTGGTCGTGGAC-3’;

Human-*GAPDH* Reverse: 5’-CCCTCCAGGGGATCTGTTTG-3’.

### Survival curves

To observe the effect of Piezo1 on survival, 40 rats were randomly divided into four experimental groups as described earlier (n = 10 per group). Survival was assessed every day until the endpoint of 7 days. Survival data were analyzed using log-rank or χ2 tests. A p-value of less than 0.05 was considered statistically significant.

### Statistical analyses

All data are presented as the mean ± SEM. The data were analyzed statistically using two-tailed, unpaired Student’s t tests and a one-way or two-way analysis of variance (ANOVA). When ANOVA showed a significant difference, pairwise comparisons between means were tested using the post hoc Tukey method (Sigma-Aldrich, Plot 12.5). *P* < 0.05 was considered statistically significant in all analyses.

## Results

### Piezo1 expression was increased in the lungs after high tidal volume mechanical ventilation

**Fig. 1:**
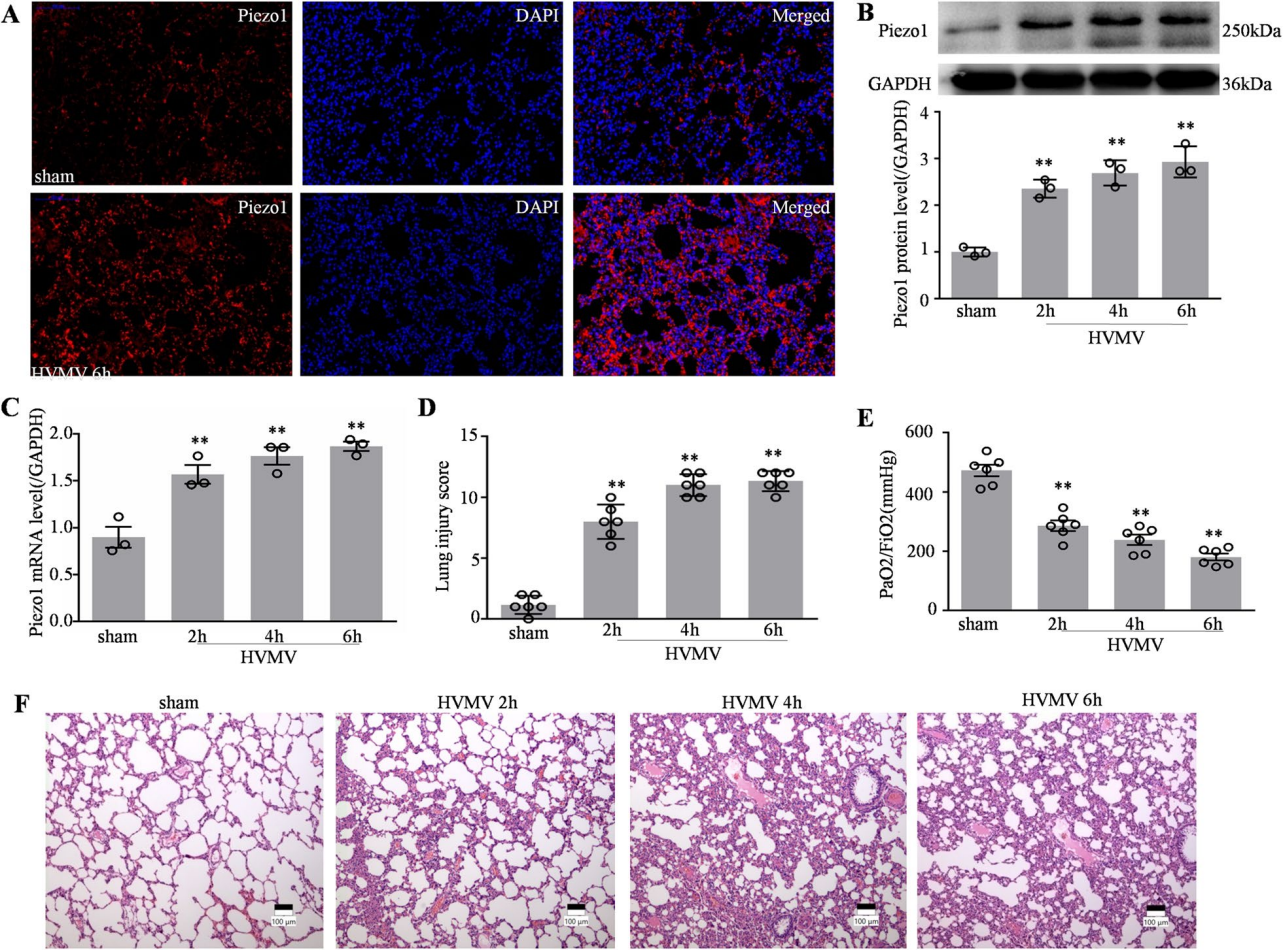
Piezo1 expression was increased in the lungs after high tidal volume mechanical ventilation. **A** Representative immunocytochemical images in rats in the sham and HVMV 6 h groups. Piezo1, and DAPI were used as markers for Piezo1 protein expression and nuclei, respectively. Scale bar = 50 μm. **B** Western blotting and quantitative analysis of Piezo1 protein levels in the above groups of rats (n = 3). **C** Quantitative analysis of *Piezo1* mRNA expression using qPCR in the above groups rats (n = 3). **D** Semi-quantitative analysis of lung tissues based on the lung injury score and **E** oxygenation index (PaO_2_/FiO_2_) in the above groups (n = 6). **F** Representative photomicrographs of lung tissues with H&E staining (original magnification × 40) in rats in sham, HVMV 2, 4, and 6 h groups. Data are shown as the mean ± SEM. **P < 0.01 versus the sham group. H&E, hematoxylin and eosin; HVMV, high tidal volume mechanical ventilation; qPCR, quantitative real-time PCR; DAPI, 4′,6-diamidino-2-phenylindole

To explore the potential role of Piezo1 in ALI, we examined whether Piezo1 expression was altered in the lung following HVMV. The expression of Piezo1 protein and mRNA increased in a time-dependent manner in the lungs at 2, 4, and 6 h after HVMV (Fig. 1B, C). Immunofluorescence images confirmed that the expression of Piezo1 in rat lung tissue increased significantly increased at 6 h after HVMV (Fig. 1A). Photomicrographs showed that, compared with that in the sham group, different degrees of lung tissue injury occurred at 2, 4, and 6 h after HVMV, and the following effects were seen: Infiltration of inflammatory cells into the lung interstitium and alveolar spaces; thickening of alveolar walls; and intra-alveolar exudation (Fig. 1F). Semi-quantitative assessment using a lung injury score demonstrated that the degree of lung injury in the HVMV groups was higher than that in the sham group (Fig. 1D), and the ratio of PaO_2_/FiO_2_ in the HVMV groups was lower than that in the sham group (Fig. 1E).

### Piezo1 expression and cell apoptosis both increased in CS-treated cells

**Fig. 2:**
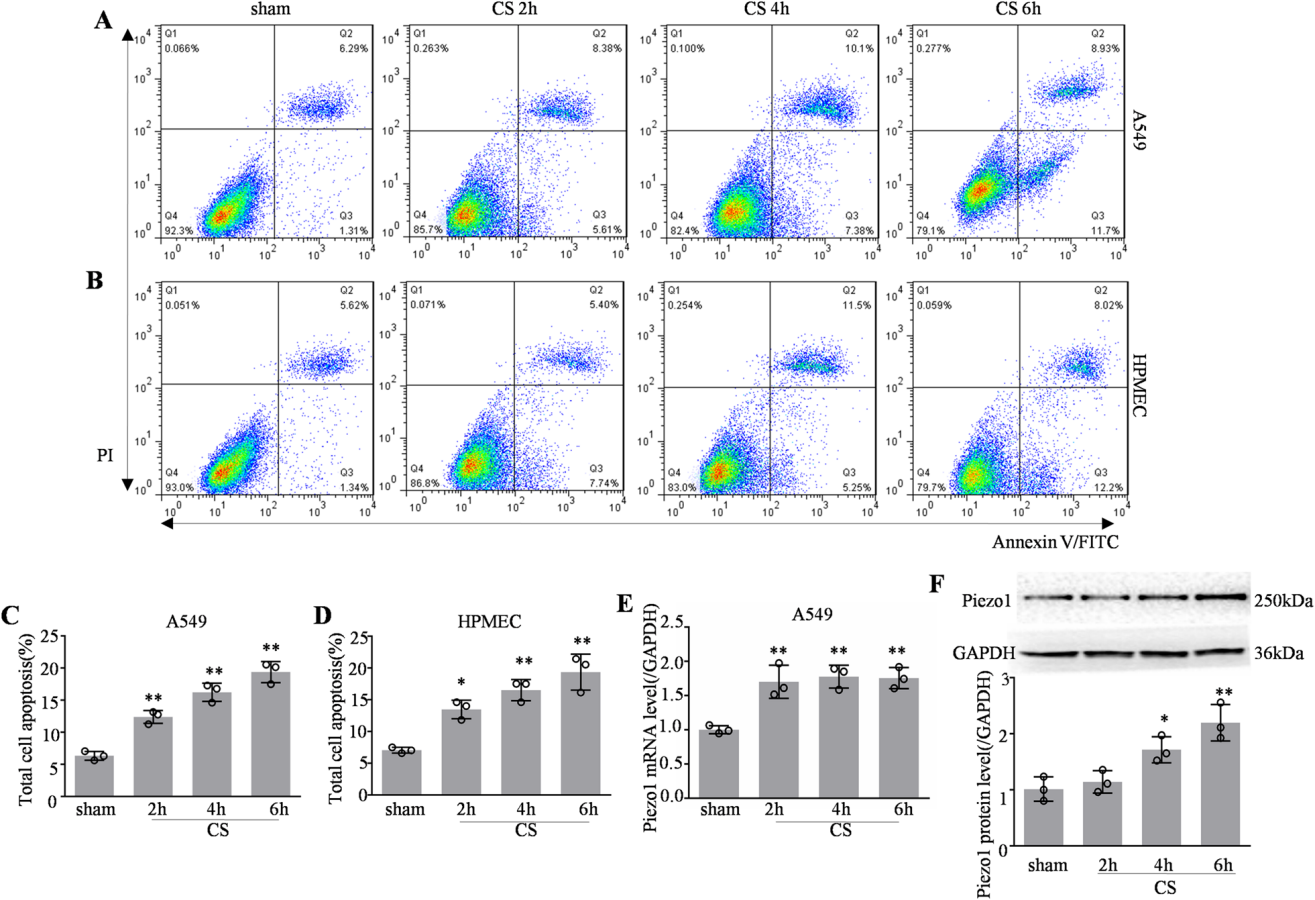
Piezo1 expression and cell apoptosis both increased in CS-treated cells. Apoptosis of A549 **A** and HPMECs **B** assessed by flow cytometry to detect Annexin V-FITC/PI staining. **C**, **D** The relative apoptosis ratio was calculated using Annexin V-positive apoptotic cells (n = 3). **E** Quantitative analysis of *Piezo1* mRNA expression by qPCR in CS-treated cells (n = 3). **F** Western blotting and quantitative analysis of Piezo1 protein levels in CS-treated cells (n = 3). Data are shown as the mean ± SEM. *P < 0.05, **P < 0.01 versus the sham group. *CS* cyclic stretch, *HPMEC* human pulmonary microvascular endothelial cell, *FITC* fluorescein isothiocyanate; *qPCR* quantitative real-time PCR

Annexin V binding and PI staining were used for cell apoptosis analysis, and a series of the representative plots of the flow cytometry analysis were presented (Fig. 2A, B). The results showed that the apoptosis rate increased in a time-dependent manner following CS treatment, and apoptosis was significantly increased in A549 cells and HPMECs compared with that in the sham group after 6 h (Fig. 2C, D). Besides, the expression of Piezo1 mRNA and protein were also time dependently increased in A549s cells after CS (Fig. 2E, F).

### Increased Piezo1 activated the RhoA/ROCK1 pathway in CS-treated HPMECs

**Fig. 3:**
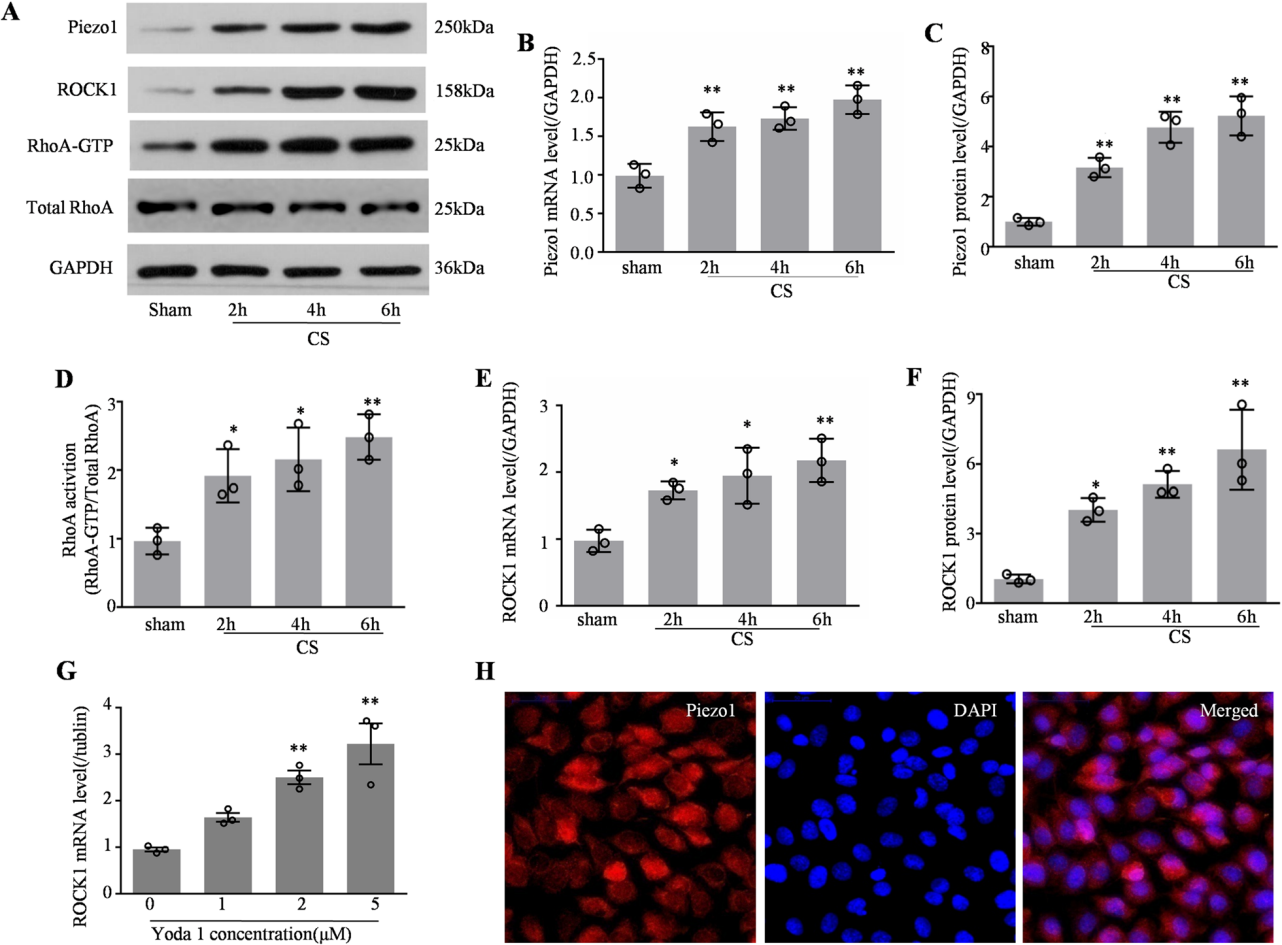
Increased Piezo1 activated the RhoA/ROCK1 pathway in CS-treated HPMECs. **A** Western blotting showing Piezo1, ROCK1, RhoA, and GAPDH levels in the sham, CS 2, 4, and 6 h cells. **B** Quantitative analysis of *Piezo1* mRNA expression using qPCR in CS-treated cells (n = 3). **C** Quantitative analysis of Piezo1 protein levels in CS-treated cells (n = 3). **D** Active GTP-RhoA was assessed using a pull-down assay after CS treatment (n = 3). **E** Quantitative analysis of *ROCK1* mRNA expression by qPCR in CS-treated cells (n = 3). **F** Quantitative analysis of ROCK1 protein levels in CS-treated cells (n = 3). **G** Quantitative analysis of *ROCK1* mRNA expression using qPCR in Yoda1-treated cells (n = 3). **H** Representative immunocytochemical images in HPMECs. Piezo1, and DAPI were used as markers for Piezo1 protein expression and nuclei, respectively. Scale bar = 50 μm. Data are shown as the mean ± SEM. *P < 0.05, **P < 0.01 versus the sham group. *CS* cyclic stretch, *HPMEC* human pulmonary microvascular endothelial cell, *qPCR* quantitative real-time PCR, *DAPI* 4′,6-diamidino-2-phenylindole

How did increased Piezo1 participate in VILI? We further examined whether Piezo1 expression was altered in HPMECs following CS. The expression of Piezo1 mRNA and protein increased in a time-dependent manner in CS-treated HPMECs (Fig. 3A–C). We also determined whether the Rho pathway might act on the CS-treated HPMECs. GTP-bound (active) RhoA in cells was measured using a RhoA activity assay, and total-RhoA was examined using western blotting. The results demonstrated that the levels of GTP-bound RhoA increased in a time-dependent manner following CS of different durations (Fig. 3A and D). Similar results were also obtained for its downstream effectors. The expression of ROCK1 mRNA and its protein increased in a time-dependent manner following CS (Fig. 3E, F). To confirm this result, we used different concentrations of Yoda1 to stimulate cells, which conformed Yoda1 as a Piezo1 selective agonist [19, 20]. To minimize the toxicity and side effects of Yoda1, a low dose of the agonist was used (~5 μM), which represented a moderate stimulus, because the EC50 of Yoda1 activation of Piezo1 is ∼25 μM [21]. As expected, the level of *ROCK1* mRNA increased in a time-dependent manner in Yoda1-treated cells (Fig. 3G). Finally, immunohistochemistry revealed the expression of Piezo1 in HPMECs (Fig. 3H).

### Blocking the increase in Piezo1 inhibited RhoA/ROCK1 pathway activation in CS-treated HPMECs

**Fig. 4:**
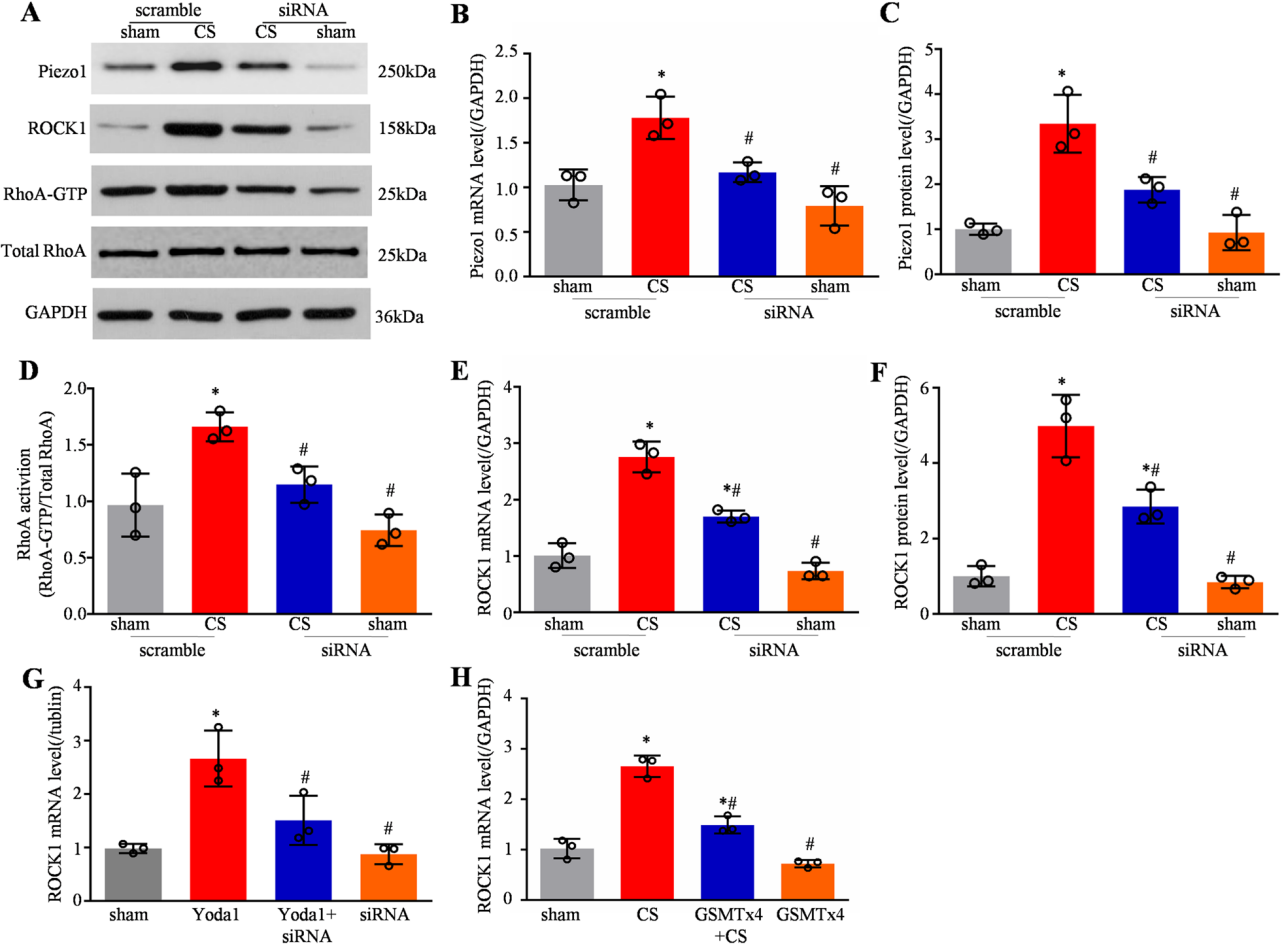
Blocking increased Piezo1 expression inhibited RhoA/ROCK1 pathway activation in CS-treated HPMECs. **A** Western blotting showed Piezo1, ROCK1, RhoA, and GAPDH levels in ad-scramble + sham, ad-scramble + CS, ad-siRNA + CS, and ad-siRNA + sham cells. Quantitative analysis of *Piezo1* **B** and *ROCK1* **E** mRNA expression using qPCR in the above cells (n = 3). Quantitative analysis of Piezo1 **C** and ROCK1 **F** protein levels in the above cells (n = 3). **D** Active GTP-RhoA was assessed using a pull-down assay in the above cells (n = 3). **G** Quantitative analysis of *ROCK*1 mRNA expression using qPCR in the sham, ad-Yoda1, ad-Yoda1 + siRNA, and ad-siRNA cells (n = 3). **H** Quantitative analysis of *ROCK1* mRNA expression using qPCR in sham, CS, ad-GSMTx4 + CS, and ad-GSMTx4 cells (n = 3). Data are shown as the mean ± SEM. *P < 0.05 versus the sham group and ^#^*P* < 0.05 versus the scramble + CS or Yoda1 group. *CS* cyclic stretch, *HPMEC* human pulmonary microvascular endothelial cell, *siRNA* small interfering RNA, *qPCR* quantitative real-time PCR

We next investigated whether blocking the CS-induced increase in Piezo1 through Piezo1-specific siRNA transfection into the HPMECs changed the RhoA/ROCK1 pathway status; a scrambled siRNA was used as a control. The level of Piezo1 mRNA and protein increased significantly in scrambled siRNA-treated HPMECs following CS for 6 h (Fig. 4A–C). However, this increase was not seen in the *Piezo1* siRNA-treated cells (Fig. 4A–C). Neither of the siRNAs altered the basal expression of Piezo1 in the sham cells. We also found that transfection with *Piezo1* siRNA affected CS-induced RhoA/ROCK1 pathway activation, as revealed by increases in the levels of GTP-bound RhoA in the scrambled siRNA-treated cells following CS for 6 h compared with those in the sham group, these increases were absent in the *Piezo1* siRNA-treated cells (Fig. 4D). And the expression of ROCK1 was not obvious in the sham or *Piezo1* siRNA cells, but it was notable after 6 h CS (Fig. 4E, F). However, the *Piezo1* siRNA transfected cells exhibited low expression of ROCK1 compared with scrambled siRNA-treated cells. Moreover, *Piezo1* siRNA pretreatment also reduced the increased ROCK1 induced by Yoda1 (Fig. 4G). For confirmation, we used GSMTx4 (an inactivated non-selective cationic MSC inhibitor) to inhibit Piezo1 activity. GSMTx4 pretreatment could effectively reduce the expression of ROCK1 induced by CS in cells (Fig. 4H).

### Inhibition of the RhoA/ROCK1 pathway did not affect the expression of Piezo1 in HPMECs

**Fig. 5:**
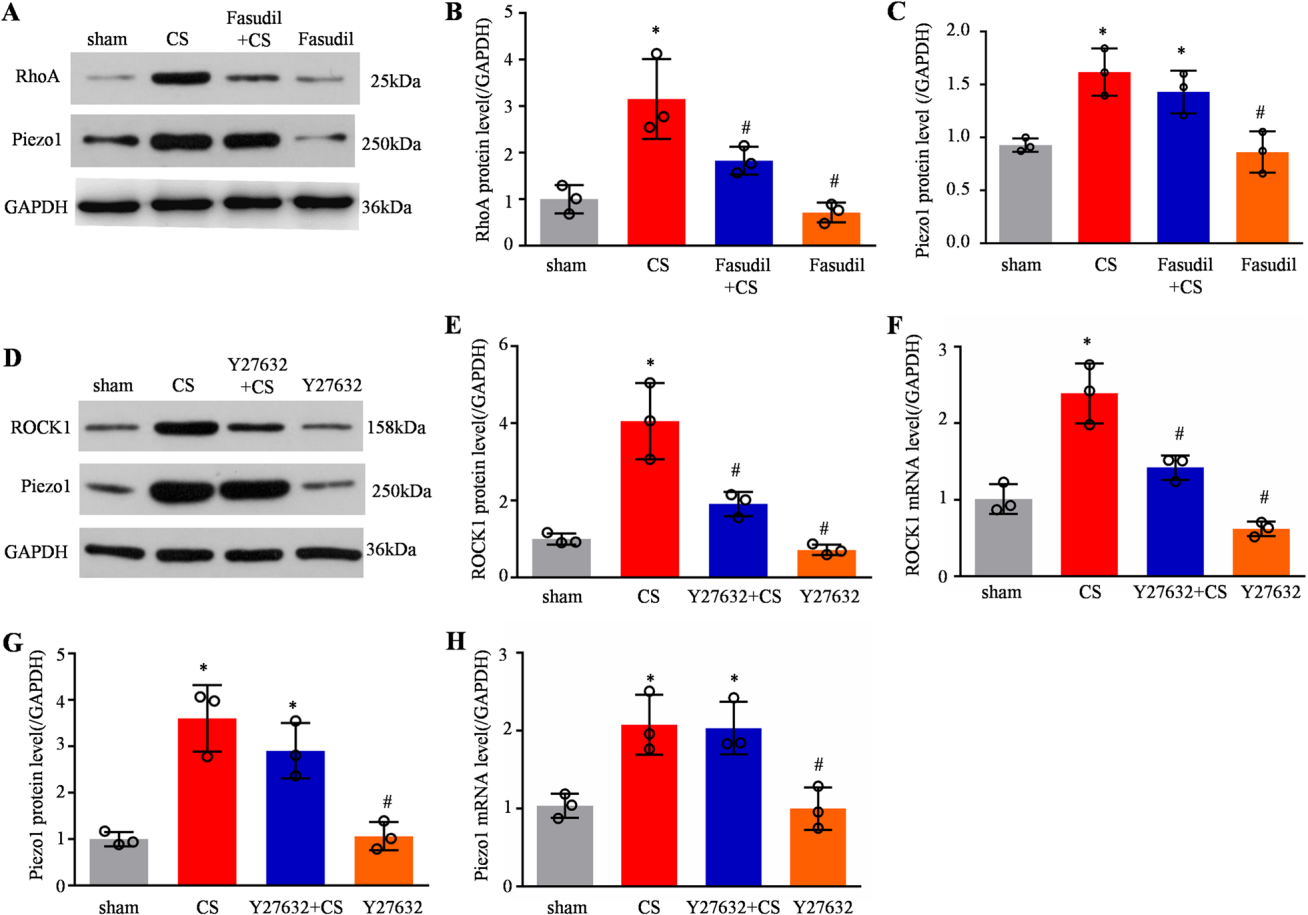
Inhibition of the RhoA/ROCK1 pathway did not affect the expression of Piezo1 in HPMECs. **A** Western blotting showed Piezo1, RhoA, and GAPDH levels in sham, CS, ad-fasudil + CS, and ad-fasudil cells. Quantitative analysis of RhoA **B** and Piezo1 **C** protein levels in the above cells (n = 3). **D** Western blotting showing ROCK1, Piezo1 and GAPDH level in sham, CS, ad-Y27632 + CS, and ad-Y27632 cells. Quantitative analysis of ROCK1 **E** and Piezo1 **G** protein levels in the above cells (n = 3). Quantitative analysis of *ROCK1* **F** and *Piezo1* **H** mRNA expression using qPCR in above cells (n = 3). Data are shown as the mean ± SEM. *P < 0.05 versus the sham group and ^#^*P* < 0.05 versus the CS group. *CS* cyclic stretch, *HPMEC* human pulmonary microvascular endothelial cell, *qPCR* quantitative real-time PCR

To further verify the possible correlation of Piezo1 and the RhoA/ROCK1 pathway, we used fasudil or Y27632, an inhibitor of the RhoA/ ROCK signaling pathway, to explore the effects of RhoA/ROCK signaling on Piezo1 overexpression. After fasudil treatment for 24 h, the increase in RhoA protein levels induced by CS was blocked (Fig. 5A, B). The expression of Piezo1 was not obvious in the sham or fasudil-treated cells, but it was significantly upregulated in CS-treated cells, with or without fasudil pretreatment (Fig. 5A, C). Similarly, after Y27632 treatment for 24 h, the increases of both ROCK1 mRNA and protein induced by CS were blocked (Fig. 5D–F). The expression of Piezo1 was not obvious in the sham or Y27632-treated cells. However, after preconditioning with or without Y27632, the expression of Piezo1 mRNA and protein were significantly upregulated in CS-treated HPMECs (Fig. 5G, H). These data indicated that Piezo1 acts as an upstream regulator of the RhoA/ROCK1 signaling pathway.

### Blocking the increased Piezo1 level attenuates VILI and improves survival in rats

**Fig. 6:**
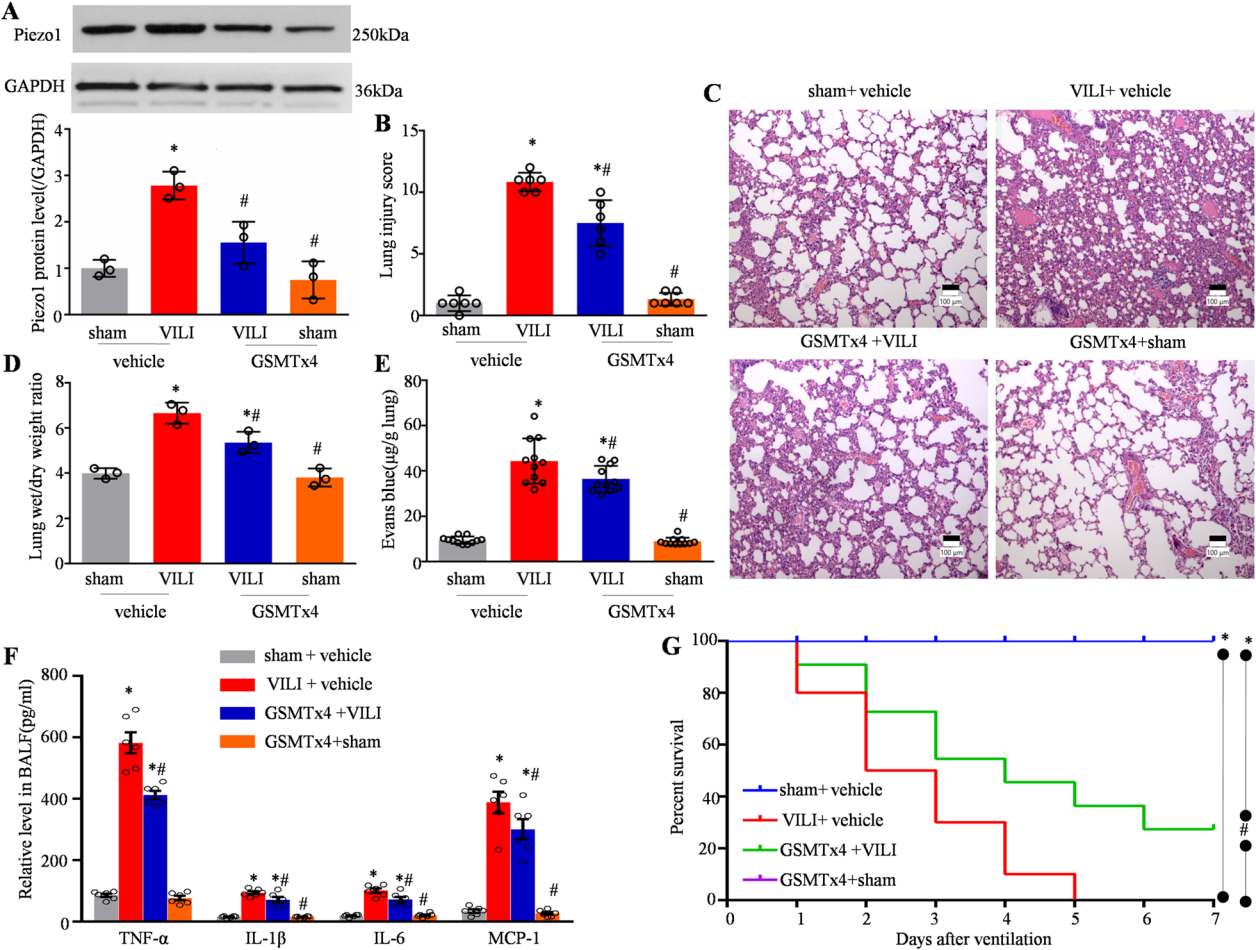
Blocking increased Piezo1 expression attenuates VILI and improves survival in rats. **A** Western blotting and quantitative analysis of Piezo1 protein levels in the above groups of rats (n = 3). **B** Semi-quantitative analysis of lung tissues based on the lung injury score (n = 6). **C** Representative photomicrographs of lung tissues with H&E staining (original magnification × 40) in rats in the vehicle + sham, vehicle + VILI, GSMTx4 + VILI, and GSMTx4 + sham groups. **D** Lung edema determined based on the wet-to-dry lung weight ratio (n = 3), and **E** protein leakage from capillaries measured by Evans blue dye extravasation in rats (n = 6). **F** The levels of TNF-α, IL-1β, IL-6, and MCP-1 in BALF, as determined by ELISA (n = 6). **G** Survival rate of rats in the above groups (n = 10). Data are shown as the mean ± SEM. *P < 0.05 versus the vehicle + sham group and ^#^*P* < 0.05 versus the vehicle + VILI group. *VILI* ventilator-induced lung injury, *H&E* hematoxylin and eosin, *BALF* bronchoalveolar lavage fluid, *ELISA* enzyme-linked immunosorbent assay

Compared with the sham group, the expression of Piezo1 in rat lung tissues was significantly upregulated after 6 h of HVMV, and pretreatment with GSMTx4 could significantly reduce piezo1 expression in lung tissues of VILI rats, and intraperitoneal administration of GSMTx4 alone did not affect its basal expression level (Fig. 6A). Photomicrographs showed that, at 6 h after HVMV, the following effects were seen: Infiltration of inflammatory cells into the lung interstitium and alveolar spaces; thickening of alveolar walls; and intra-alveolar exudation (Fig. 6C). However, GSMTx4 preconditioning attenuated these histological changes. Semi-quantitative assessment using a lung injury score demonstrated that the degree of lung injury in the VILI + GSMTx4 group was lower than that in the VILI + vehicle group (Fig. 6B). The lung wet-to-dry weight ratio increased significantly at 6 h after VILI administration (Fig. 6D). Extravasation of Evans Blue Dye showed that VILI induced a significant increase in leakage into the lung (Fig. 6E). When the animals were pretreated with 10 μg GSMTx4, lung edema and capillary leakage were reduced significantly at 6 h after VILI administration (Fig. 6D, E). GSMTx4 alone did not affect these variables in the sham rats. Six hours after HVMV, BALF was collected and the concentration of inflammatory cytokines in BALF was detected using ELISA. The results showed that the concentration of pro-inflammatory cytokines, e.g., TNF-α, IL-1β, IL-6, and MCP-1, increased significantly in lung tissue (Fig. 6F). However, after GSMTx4 pretreatment, the concentration of TNF-α, IL-1β, and IL-6 in BALF decreased significantly (Fig. 6F). These findings indicated that blocking Piezo1 could alleviate the inflammatory reaction of lung tissue in VILI rats.

As shown in Fig. 6G, the survival rate of rats in the sham group was 100%, and the survival rate was significantly decreased in the VILI group compared with that in the sham operation group. The survival rate after HVMV was 50% on the second day, which decreased to 0% on the fifth day. Pretreatment with GSMTx4, however, improved the survival rate to 70% on second day and 30% on day 7, and at least 30% of the rats survived. This result confirmed the protective effect of blocking Piezo1 on mortality in rats with VILI.

## Discussion

Mechanical ventilation can both provide respiratory support to patients with ALI and aggravate pre-existing lung injury, prompting the progression of ALI to ARDS, as well as increasing patients’ mortality. In recent years, some scholars have proposed and developed a lung protective ventilation strategy using low tidal volume combined with lung recruitment and PEEP as the main component, which achieved certain effects while also increasing the risk of diaphragmatic function barriers [22, 23]. Further understanding of the pathogenic mechanisms of VILI may provide a new avenue for the management of this disorder. In the present study, we demonstrated that Piezo1 participates in the mechanisms of VILI in rats and CS-induced cell apoptosis by activating RhoA/ROCK1 signaling in rats.

Traction of the alveoli by large tidal volumes is an important etiology of VILI, with distortion of the alveolar epithelium versus the pulmonary endothelium from stress, both of which are mechanically damaged when high tidal volumes are ventilated. More importantly, the pulmonary vascular endothelium is subjected to mechanical stretch leading to increased cell membrane permeability; intravascular exudation of substances such as albumin and erythrocyte debris into the pulmonary interstitium; and products such as phospholipase released by neutrophils and macrophages after activation, which can interfere with and inactivate alveolar surfactant, thereby affecting alveolar function [24]. Given that MV can activate Piezo1 channels in the lungs of ARDS rats, which increased the intracellular Ca^2+^ content in alveolar epithelial cells, downregulated the expression of anti-apoptotic protein Bcl-2, and increased alveolar epithelial cell apoptosis [25]. Further studies showed that increasing pulmonary vascular hydrostatic pressure in mice, either using aortic constriction or elevating the left atrial pressure, resulted in severe pulmonary edema after disruption of the pulmonary vascular barrier in wild-type mice, whereas specifically knocking out *Piezo1* in pulmonary endothelial cells significantly reduced pulmonary vascular permeability and the extent of pulmonary edema in mice. In addition, degradation of adherens junction proteins VE-cadherin, β-catenin, and p120-catenin was not significant [26]. In the present study, we found that HVMV significantly induced pulmonary interstitial edema, alveolar wall thickening, and destruction of alveolar morphology in rat lung tissue, and the injury was more pronounced and the oxygenation index was significantly reduced as the duration of MV increased. During this process, Piezo1 expression in rat lung tissue also showed significant upregulation with increased ventilation time. By immunohistochemical staining, we found that Piezo1 was widely expressed in rat lung tissue. Alveolar epithelial and endothelial cells, the earliest effector cells to appear alterations within the lung, bear the brunt of the damage when ALI is initiated. In this study, we found that after massive mechanical stretching of the lung epithelium and endothelium (20%) [27], mimicking VILI in vitro, both the epithelium and endothelium were significantly damaged and the apoptosis rate increased in a time-dependent manner. Consistent with the injury, Piezo1 expression also showed time-dependent upregulation, suggesting that upregulated *Piezo1* gene expression in the lung might be an important risk factor for the pathogenesis of VILI. This could be related to the fact that Piezo1 acts as a nonselective cation channel and is permeable to extracellular Ca^2+^ influx [28]. Intracellular Ca^2+^ acts as a second messenger that can activate different downstream biochemical signaling pathways and biological effects. It reported that loading mice with ex vivo perfused lungs with high peak inspiratory pressure (PIP) ventilation increased their pulmonary vascular barrier permeability, and that reducing Ca^2+^ influx caused by channel activation, by means of pretreatment with TRPV4 inhibitors or gene knockout, could partially abolish pulmonary edema resulting from disruption of the air-blood barrier [29]. In addition, high PIP ventilation can activate TRPV4 channels on the surface of alveolar macrophages to trigger Ca^2+^ signals, leading to a large production of NO and O^2−^, which causes oxidative damage [30]. Using real-time Ca^2+^ imaging, we observed that acute elevation of airway pressure in healthy mice induced a significant increase in free Ca^2+^ in endothelial cells that lasted for more than 15 min.

Alveolar capillary barrier dysfunction is one of the important pathological features of ALI [31]. The alveolar capillary barrier compositional structure includes pulmonary microvascular endothelial cells and alveolar epithelial cells, either of which, when damaged, affects the homeostasis of lung function. However, endothelial cells are the first defense barrier, and when inflammatory injury occurs, pulmonary microvascular endothelial cells are first damaged, and cell permeability rapidly increases, causing capillary leakage. Therefore, we further explored how Piezo1 plays a role in VILI and its possible downstream regulatory mechanisms by mechanically stretching HPMECs. Previous studies have reported that the RhoA/ROCK pathway, an intracellular signaling pathway, is involved in the development of ALI induced by LPS [32, 33]. Similarly, our previous study also found abnormal accumulation of ROCK1 protein and increased expression of *RhoA* and *Rock2* mRNA in the rat lung under exogenous endotoxin stimulation. Significantly higher mean optical density values of pulmonary perivascular ROCK1 protein were observed in rat lung sections. Small tidal volume ventilation reduces the degree of early lung injury in LPS rats, and the reason may be related to the inhibition of the RhoA/ROCK1 signaling pathway [9]. In vitro studies have found that CS at a strain of 15% activates RhoA through the protein kinase activated receptor 1 pathway, causes cytoskeletal rearrangements, forms actin tension filaments, and increases endothelial permeability [34, 35]. In this study, we showed that a strain of 20% on periodically pulled endothelial cells showed a time-dependent upregulation of Piezo1 expression, along with the activation of the intracellular RhoA/ROCK1 signaling pathway. The expression of ROCK was also upregulated in a time-dependent manner. Interestingly, pretreatment with different concentrations of Yoda1 (a Piezo1 selective agonist [19]) in cells activated Piezo1 channel activity, and the expression of ROCK1 also appeared to be significantly upregulated, thus Piezo1 might act as an upstream regulatory molecule of the RhoA/ROCK1 signaling pathway. To further test this hypothesis, we pretreated cells with siRNA to knockdown *Piezo1* expression and found that the RhoA/ROCK1 signaling pathway was significantly inhibited, and the expression of ROCK1 also decreased significantly. Similarly, pretreatment with *Piezo1* siRNA caused downregulation of ROCK1 expression in the presence of pre-activated cellular Piezo1 channel activity. We also further confirmed this result using GSMTx4, an endogenous cation channel inhibitor [36]. Conversely, when we pretreated endothelial cells with fasudil and Y27632 [37], which inhibit RhoA and ROCK1 protein expression, Piezo1 expression did not show significant alterations, thus, Piezo1 might participate in VILI through the downstream activation of the RhoA/ROCK1 signaling pathway.

The Rho family of small G proteins is an important molecule in the regulation of intercellular adherens junctions and intracellular actin junctions, and plays an important regulatory role in pulmonary vascular endothelial barrier function [38]. A large amount of Ca^2+^ influx causes the intracellular Ca^2+^ concentration to become too high, which causes an inflammatory response and disrupts intercellular junctional junctions; and the Ca^2+^ influx also significantly upregulates Rho GTPase activity [39, 40]. By contrast Piezo1 is a bona fide mechanosensitive ion channel protein in mammals and allows Ca^2+^ passage, and mediates remodeling of the cytoskeleton and stress alterations, representing a key element of the mechanotransduction process [41–43]. This may partly explain why Piezo1 is able to regulate the RhoA/ROCK1 signaling pathway and is involved in ventilator-associated lung injury.

In the present study, ventilator-associated lung injury was confirmed by histological analysis, in which HVMV was performed to induce ALI. In keeping with the pathogenesis of VILI, HVMV was confirmed by an increase in the water content and protein leakage in the lungs. GSMTx4 was used to inhibit Piezo1 channel activity, which significantly attenuated these abnormalities, indicating the therapeutic role of Piezo1 in VILI in rats. Our data demonstrate that pulmonary levels of pro-inflammatory cytokines increased markedly in rats that underwent HVMV, and that Piezo1 inhibition resulted in a decrease in the accumulation of these cytokines. Furthermore, although the 7-day survival rate of rats in the GSMTx4 group was not different from that in the sham group, inhibition of Piezo1 ultimately resulted in an improvement in the overall survival rate of the model rats. Thus, these findings are consistent with the above data.

We must acknowledge the limitations of our study. One of the limitations was that the observation period was limited to 6 h, and for several chemicals involved in this study, the use of a larger concentration range was not adopted. Moreover, indicators of cellular electrophysiology were not assessed in this study, such as the ability to observe the concentration changes and flow of intracellular and extracellular Ca^2+^ in real time, which would have improved this study and thus require further investigation.

## Conclusions

Using an HVMV-induced model of VILI in rats, we demonstrated that Piezo1 might have a role in VILI-induced pathological changes and apoptosis of endothelial and epithelial cells; the water content and protein leakage in lungs; the induction of systemic inflammatory mediators; and the 7-day mortality rate in rats. Furthermore, the results of the molecular analysis indicated that Piezo1 contributes to VILI by activating RhoA/ROCK1 in rats. Thus, Piezo1 might represent an effective therapeutic agent for the treatment of lung injury.

## Data Availability

The datasets used and/or analyzed during the current study are available from the corresponding author on reasonable request.

## Abbreviations

MV: Mechanical ventilation
ALI: Acute lung injury
ARDS: Acute respiratory distress syndrome
HVMV: High tidal volume mechanical ventilation
PEEP: Positive end-expiratory pressure
CS: Cyclic stretch
BALF: Bronchoalveolar lavage fluid
HPMEC: Human pulmonary microvascular endothelial cell
PCR: Polymerase chain reaction
ELISA: Enzyme-linked immunosorbent assay
PIP: Peak inspiratory pressure
